# Thirty-day mortality rates among young adult stroke patients and their characteristics at Kiruddu and Mulago hospitals in Uganda: A prospective observational cohort study

**DOI:** 10.1371/journal.pgph.0001892

**Published:** 2023-10-26

**Authors:** Adrian Mwota Nampogo, Abdu Kisekka Musubire, Peace Bagasha, Scovia Mbalinda, Shirley Moore, Elly. T. Katabira, Martha Sajatovic, Mark Kaddumukasa

**Affiliations:** 1 Department of Medicine, School of Medicine, College of Health Sciences, Makerere University, Kampala, Uganda; 2 Kiruddu National Referral Hospital, Kampala, Uganda; 3 Department of Nursing, College of Health Sciences, Makerere University, Kampala, Uganda; 4 Frances Payne Bolton School of Nursing, Case Western Reserve University, Cleveland, OH, United States of America; 5 Neurological and Behavioral Outcomes Center, University Hospitals Cleveland Medical Center & Case Western Reserve University School of Medicine, Cleveland, OH, United States of America; PLOS: Public Library of Science, UNITED STATES

## Abstract

Stroke outcomes among young adults in Uganda are unclear. This study therefore determined the clinical characteristics and 30-day outcome among young adults with an acute stroke. In a prospective observational cohort study, 61 young adults with confirmed stroke were followed up for 30 days. Socio-demographic and clinical characteristics were collected using a study questionnaire. Kaplan–Meier curves, and modified Poisson regression were performed for factors associated with the 30-day mortality outcome. A third of the screened stroke survivors, (61/195) were young adults aged between 18 and 50 years. About two-thirds were male. More than half were diagnosed with ischaemic strokes while 42.6% had a haemorrhagic stroke. Nearly half (29/61) were known hypertensives, 43% (26/61) had a history of alcohol consumption with 95% classified as dependent on CAGE assessment. Ten percent had a prior smoking history while 29% of the female gender had a prior history of oral contraception use. Twenty-three percent (14/61) of the young stroke patients died within 30 days of stroke onset (95% CI: 0.01, 0. 901). A history of smoking (adjusted prevalence ratio: aPR;5. 094, 95% CI: 3.712, 6. 990) and stroke severity National Institutes of Health Stroke score (NIHSS) >16; Prevalence ratio (PR) -3. 301, 95%CI: 1. 395, 7. 808) and not drinking alcohol (aPR (adjusted prevalence ratio) -7. 247, 95% CI: 4. 491, 11.696) were associated with 30- day mortality. A third of all stroke survivors were young adults. About 23. 3% died within 30 days of stroke onset. Stroke severity and a history of smoking were associated with mortality. Identifying high risk patients and early outpatient follow up may help reduce the 30-day mortality in our settings.

## Introduction

Stroke is the second leading cause of mortality worldwide and a rising health care problem [1]. While stroke is considered a disease of middle-aged and elderly patients, an emerging trend of increasing strokes in younger adults has been observed [2–4]. This trend might be resulting from the escalating burden of classical vascular and other risk factors like hypertension, diabetes, obesity, hypercholesterolemia, sickle-cell disease, rheumatic heart disease, and HIV are significantly prevalent among young adults in Uganda [5–11].

Young adults with stroke, tend to suffer severe forms of stroke and have an associated higher mortality [12]. Few studies have emphasized stroke outcomes among the young adults in Uganda. Earlier studies among stroke patients have reported high mortality rates ranging from 18.4% to 41% among those aged more than 50 years [13, 14]. Drivers of mortality among young adults with stroke have not been extensively studied as most studies look at the general population of stroke. Understanding the associated factors and outcomes of stroke among young adults urgently is needed to devise interventions. Therefore, we determined the 30-day mortality outcome and associated factors among young adults with stroke in our setting.

## Materials and methods

### Study design

Between March and June 2022, a prospective observational study was conducted to enrol patients who had experienced a stroke. Stroke was defined as an acute episode of acute neurological dysfunction presumed to be caused by ischemia or haemorrhage, persisting for more than 24 hours [15] and presenting to hospital within a period of seven days. The patients aged 18–50 years were then monitored daily for a period of 30 days. These patients were then monitored for a duration of 30 days. The study was conducted at both Kiruddu Referral hospital and Mulago Hospital. Kiruddu hospital is a 170-bed capacity facility, approximately 13 kilometers from the city center while Mulago National Referral Hospital is a 1650-bed capacity facility located within the city center. These two hospitals serve as teaching hospitals for Makerere College of Health Sciences. They have no specialised stroke units within Uganda though stroke patients are managed in general neurology units under the care of neurologists. Both sites functional computerized tomography radiological services for stroke diagnosis. Mulago follows the Uganda clinical guidelines 2016 for stroke and hypertension treatment.

### Study population

We enrolled patients who provided written informed consent and fulfilled the study inclusion criteria below; 1) aged between 18 to 50 years, 2) a confirmed diagnosis of an acute stroke by computerized tomography (C.T.) brain scan by Siemens SOMATOM CTscan; Germany 3) an acute stroke presenting within seven days of symptom onset, and 4) provision of written informed consent. We excluded stroke patients who were unconscious patient with no proxy to provide vital information.

### Sample size

An estimated sample size of 62 young stroke patients was enrolled. Based on a mortality rate among young adults with stroke was 3.8% using Kish Leslie’s formula while adjusting for a non-response of 10% [16]. This was the biggest sample size for the proportion of stroke patients aged 18–50 years thus and was utilized for the secondary objective that looked at the 30-day mortality outcome among these study participants.

### Data collection

A pre-tested and standardized questionnaire was used as a data collection tool by the study team. The following study variables were collected; socio-demographics such as age, sex, occupation, lifestyle risk factors (alcohol, smoking, physical activity) and clinical symptoms, blood pressure, temperature, type of stroke and date of symptom onset were collected. Comorbidities like hypertension, diabetes, HIV, sickle-cell disease, known rheumatic heart disease, were ascertained. Blood pressure was measured using a digital blood pressure machine (Omron 5 series). For patients who were too weak to sit up, blood pressure measurement was taken in supine position. For those able to sit, it was taken in the sitting position. The two blood pressure measurements were taken at an interval of 5 min and the average blood pressure recorded as the final blood pressure. Based on JNC8, we considered individuals with systolic blood pressure (SBP) ≥140 mmHg or diastolic blood pressure (DBP) ≥90 mmHg as hypertensive [17]. Diabetes was defined as having a glycated haemoglobin level of > 6.5% or fasting blood glucose of greater than 7.0mmol/l [18].

Current medications such as anti-hypertensive, anti-platelet drugs, anti-coagulants, oral or injectable contraceptives, lipid lowering drugs, and anti-diabetic drugs and Laboratory characteristics like full blood count performed by a Sysmex X10-L550 CBC machine; Germany., HIV serology, fasting blood glucose were recorded. Stroke severity was determined using the National Institute of Health Stroke Scale (NIHSS) which is an objective clinical tool used in the evaluation of stroke severity [19]. It comprises of 15 neurologic examination items assessed like level of consciousness, language, neglect, visual field loss, extra ocular movement, motor strength, ataxia, dysarthria and sensory loss. Low scores indicate less severe strokes while high scores indicate severe strokes.

Functional disability was determined using the modified Rankin Scale (mRS). The mRS is a measure of functional ability or dependence in daily life and has been widely used as a measure of global disability in patients with stroke [20]. The study participants were followed up during the admission period until they were discharged from hospital. For those who were discharged before attaining day 30 post-stroke, we conducted a follow up visit to determine the study outcomes.

### Quality control

The data collection tool was pre-tested before the start of the study. The study team was trained on data collection form completion. The data collection forms were double-checked and ensured proper filling at the end of each day.

### Statistical analysis

The patients’ baseline demographic and clinical factors were summarized in tables using proportions and frequencies for categorical data. The continuous variable (age) was summarized using the median and interquartile range because the data was not normally distributed. Socio-demographic, clinical, and laboratory characteristics by stroke type were compared using the chi-square test or fisher’s exact test. Modified Poisson regression was used to determine factors associated with mortality among young adult stroke patients since the proportion of participants that died was more than 10%. Clustered robust standard errors were used to adjust for clustering in the data. Bivariate analysis was performed to assess the association of independent variables with mortality, and only variables with p-value <0.05 were included in the multivariable model because of the limitation in the sample size. Multivariable analysis was performed to determine factors associated with mortality among young adults with stroke. A stepwise method was done including all clinically significant variables, and all variables with p-values greater than 0.05 were dropped by backward removal. Product terms were formed between the variables with p-value < 0.05 and the assessment of interaction was done using the chunk test. Assessment of confounding was done by bringing back the basic variables that were dropped starting with the variable that was dropped last. A percentage change of more than 10% meant there was confounding. All the confounders were left in the final model and these included; socio-demographics, age, sex, occupation, address, religion, marital status, level of education, lifestyle risk factors (alcohol, smoking, physical activity). The goodness of fit test was performed using the Hosmer Lemeshow test. The possible associations between mortality and other independent variables were considered statistically significant at p-value <0.05 or 95% confidence intervals that did not contain the null value. Kaplan–Meier curves were used to show the survival probability of young adults with stroke after 30 days and the log-rank test was performed to check if the curves were statistically different at a set level of significance alpha <0.05%.

### Ethical considerations

This study was conducted under the ethical principles stated in the Declaration of Helsinki (1996), the National Guidelines of Research Involving Humans and Research Participants, and the applicable guidelines on Good Clinical Practice. The School of Medicine Research and Ethics Committee (SOMREC) approved the study under REC number Mak-SOMREC-2021-230. Administrative clearance was acquired from both Kiruddu and Mulago National referral Hospitals. The study objectives were explained to the study participants, who were assured of confidentiality through anonymous data storage and reporting.

## Results

### Profile of the study

**Fig 1:**
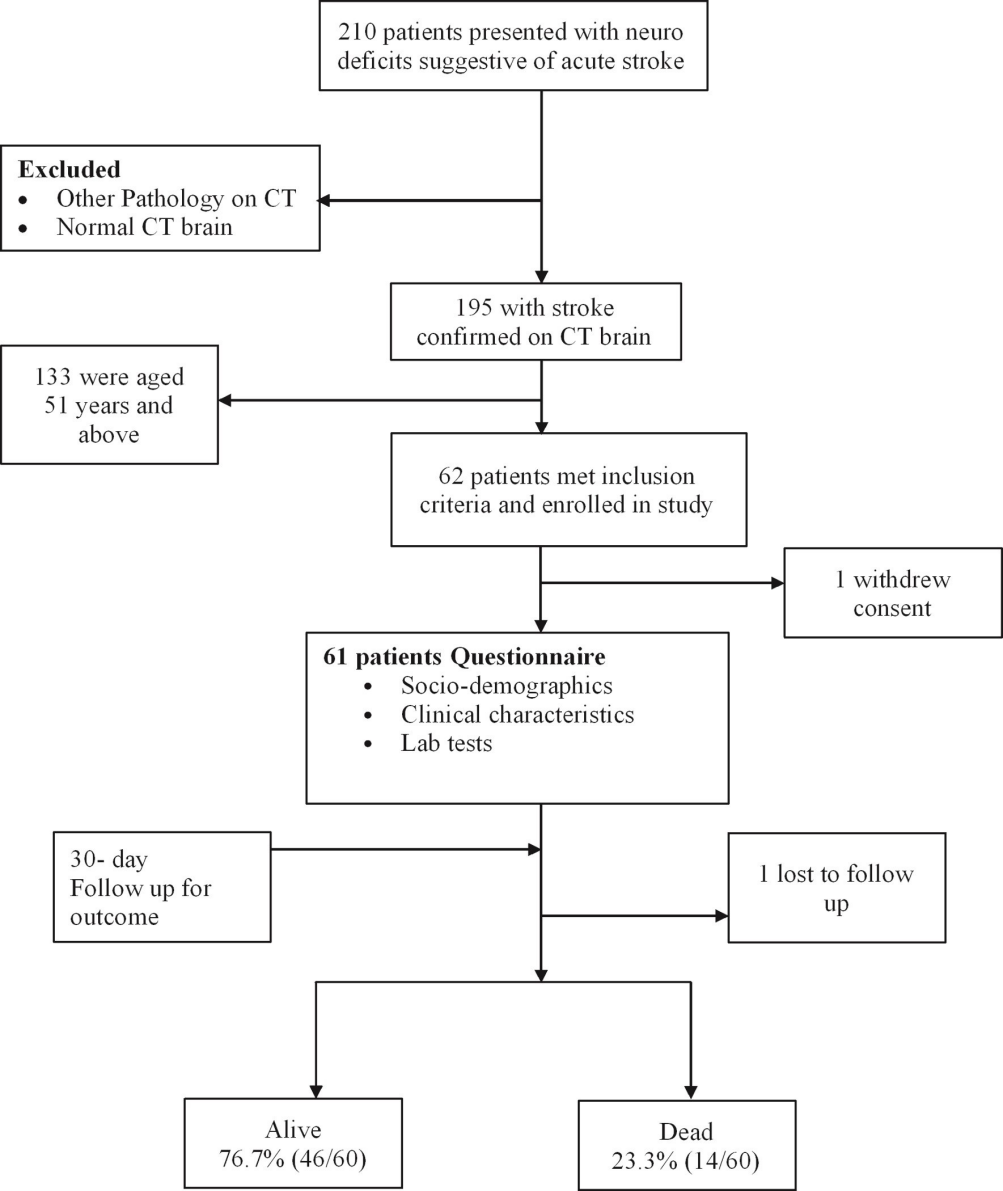


In this study, 195 patients with confirmed acute stroke presented to both Kiruddu and Mulago hospital emergency units between 28^th^ March 2022 and 19^th^ June 2022. Sixty-one study participants met the study inclusion criteria and were enrolled. One patient was lost to follow-up within the 30-day period. The proportion of young adults with confirmed stroke was 31.3%, (61/195); (95% CI: 23.5%, 39.1%), as shown in “Fig 1”.

### Socio-demographic characteristics

Of the 61 patients enrolled, the proportion of young adults with stroke at Mulago Referral and Kiruddu Referral hospitals was 31.3% (95% CI: 23.5%, 39.1%. The median age (IQR) was 42, (37–47) years. Most participants were male, 65.6% (40/61), 72.1% (44/61) were married, and 65.6% (40/61) were employed. Among the young adults with stroke, 9.8% (6/41) had a positive history of smoking, 42.6% (26/41) had a positive history of alcohol consumption, and of these, 95% were dependent with a CAGE score of ≥1. Other socio-demographic characteristics are shown in Table 1.

**Table 1:** Socio-demographic characteristics of 61 young adults with stroke.

Variable	Overall N = 61(%)	Haemorrhagic N = 26 (%)	Ischaemic N = 35 (%)	P-value
Age (years)				
18–30	9 (14.8)	1 (3.9)	8 (22.9)	0.115
31–40	16 (26.2)	8 (30.8)	8 (22.9)	
41–50	36 (59.0)	17 (65.4)	19 (54.2)	
Sex				
Female	21 (34.4)	7 (26.9)	14 (40.0)	0.288
Male	40 (65.6)	19 (73.10	21 (60.0)	
Tribe				
Ganda	24 (39.3)	16 (61.5)	21 (60.0)	0.903
Others	37 (60.7)	10 (38.5)	14 (40.0)	
Marital status				
Married	44 (72.1)	23 (88.5)	21 (60.0)	0.014
Single	17 (27.9	3 (11.5)	14 (40.0)	
Occupation				
Unemployed	21 (34.4)	8 (30.8)	13 (37.1)	0.604
Employed	40 (65.6)	18 (69.2)	22 (62.9)	
Level of education Primary level	27 (44.3)	10 (38.5)	17 (48.6)	0.432
Above primary level	34 (55.7)	16 (61.5)	18 (51.4)	
Smoking status				
Yes	6 (9.8)	1 (3.9)	5 (14.3)	0.227
No	55 (90.2)	25 (96.1)	30 (85.7)	
Alcohol consumption				
Yes	26 (42.6)	11 (42.3)	15 (42.9)	0.966
No	35 (57.4)	15 (57.7)	20 (57.1)	
Family history of stroke				
Yes	10 (17.5)	4 (15.4)	6 (17.1)	0.854
No	47 (82.5)	22 (84.6)	29 (82.9)	

### Clinical and laboratory characteristics

The majority, 59% (36/61) of the participants, had presenting complaints lasting less than three days. The majority, 57.4% (35/61), had ischaemic strokes on head C.T. scan and 42.6% (26/61) had haemorrhagic strokes. About 41% (25/61) had elevated pressures at admission (Systolic Blood Pressures >140mmhg), while all the diastolic blood pressures for all study participants were within normal ranges. Only 10.2% (6/61) were HIV seropositive, and 17.5% (10/61) had a positive family history of stroke.

More than half of the study participants (33/61), had a National Institutes of Health Stroke Scale (NIHSS) score >16 and 73.8% (45/61) had a significant disability of 4–6 by modified Rankin score (mRs) at admission. About 11.4% (7/61) of the participants were in coma (GCS<8). More than half, 55.4% (34/61), had comorbidities, and hypertension was the leading comorbidity with 47.6% (29/61). About 16.4% (10/61) of the young adults with stroke had multiple comorbidities. About 28.6% (6/21) of the females had oral contraception use history. Among the 61 study participants, 27 (44.3%) were receiving lipid lowering drugs, 5 (8.2%) oral hypoglycaemic agents, 8 (13.1%) anti-platelets and 10 (28.6%) were receiving anti-coagulants, see Table 2.

**Table 2:** Clinical and laboratory characteristics of study participants.

Variables	Overall n = 61(%)	Haemorrhagic n = 26 (%)	Ischaemic n = 35 (%)	p-value
**Presenting complaint (days)**				0.979
≤ 3	36 (59.0)	14 (53.9)	22 (62.9)	
>3	25 (41)	12 (46.1)	13 (37.1)	
**Temperature**				0.772
Afebrile	48 (78.7)	20 (76.9)	25 (80.0)	
Febrile	13 (21.3)	6 (23.1)	7 (20.0)	
**GCS**				0.472
15	17 (22.9)	6 (23.1)	11 (31.4)	
<15 (altered)	44 (72.1)	20 (76.9)	24 (68.6)	
**Blood pressure**				0.078
SBP>140	25 (41)	14 (53.9)	11 (31.4)	
SBP ≤140	36(59)	12 (46.1)	24 (68.6)	
**Disability (mRS)**				0.629
0–3 (mild -moderate)	16 (26.2)	6 (23.1)	10 (28.6)	
4–6 (severe)	45 (73.8)	20 (76.9)	25 (71.4)	
**Stroke severity (NIHSS)**				0.627
0–15 (mild- moderate)	28 (45.9)	11(42.3)	17 (48.6)	
16–42 (severe)	33 (54.1)	15 (57.7)	18 (51.4)	
**RPR test (35)** *				1.000
Reactive	2 (5.7)	1 (6.7)	1 (5.0)	
Non–reactive	3 (94.3)	14 (93.3)	19 (95.0)	
**HIV (n = 59)** *				1.000
Reactive	6 (10.2)	2 (8.0)	4 (11.8)	
Non–reactive	33 (94.3)	23 (92.0)	30 (88.2)	

*n = number of results returned.

### 30-day mortality outcome of the study participants

**Fig 2:**
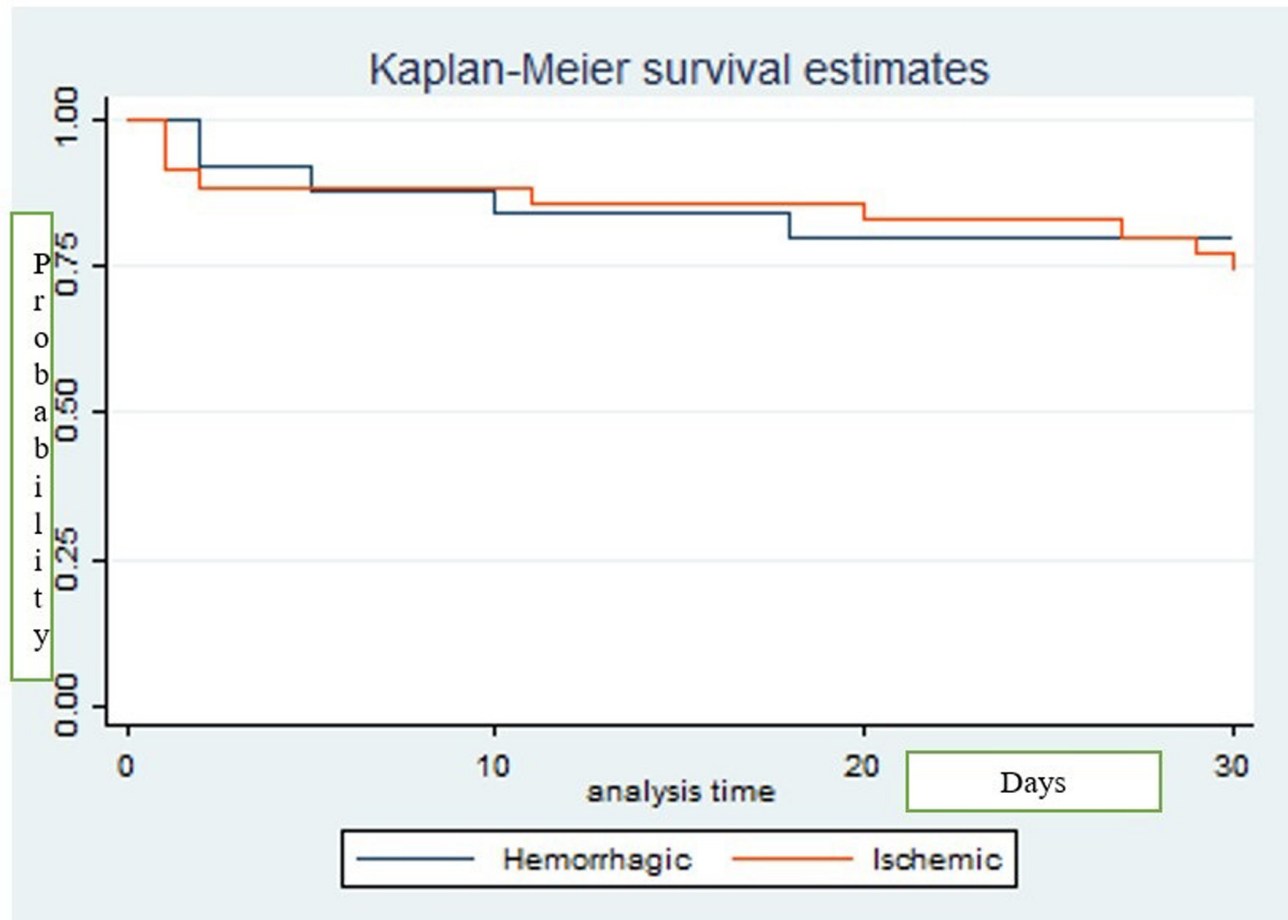


Of the 61 study participants enrolled, 98.4% (60/61) were followed to the 30^th^ day from stroke onset, with 23%, 14/60 of the study participants having died on day 30 of follow up. The survival probability of patients with ischaemic stroke 0.743 (95% CI: 0.564, 0.857) was better than the survival probability of patients with haemorrhagic stroke 0.801 (95% CI: 0.598, 0.915), as shown in the Kaplan–Meier curve see “Fig 2”, showing the mortality differences in haemorrhagic and ischemic strokes. The survival probability of haemorrhagic stroke was better by day 30 compared to ischemic stroke although it was not statistically significant with a p-value of 0.26. From the Kaplan–Meier survival curve, most patients died within the first ten days.

### Factors associated with mortality among the participants

In the bivariate analysis of socio-demographic characteristics: being single prevalence ratio (PR) of 1.405, 95% CI: 1.362, 1.450), smoking (PR; 0.150, 95%CI: 1.082, 2.079), and not taking alcohol (PR;2.619, 95% CI: 1.505, 4.557) were significantly associated with 30-day mortality. However, at multivariable analysis, smoking, not drinking alcohol and having a high NIHSS stroke severity score of >16 was associated with mortality with adjusted prevalence ratios (aPR) of 5.094;95% CI:3.712,6.990), (aPR: 7.247; 95%CI: 4.491, 11.696), and (aPR;3.301, 95%CI:1.395, 7.808) respectively. Being single (aPR;0.785, (5%CI: 3.712, 6,990) and having an ischaemic type of stroke (aPR;1.473, 95% CI: 0.804–2.700) were not associated with mortality. Details of the association of 30-day mortality with socio-demographics, clinical and laboratory characteristics are shown in Tables 3 and 4.

**Table 3:** Socio-demographic and clinical characteristics of study participants associated with 30-day stroke mortality at bivariate analysis.

Variable	Alive n = 46(%)	Dead n = 14(%)	crude PR	p-value
**Age (years)**				
≤30	7(15.2)	2(14.3)	1.000	**1.000**
31–40	11(23.9)	4(28.6)	1.200	
>41	28(60.9)	8(57.1)	1.000	
**Sex**				
Female	15(32.6)	5(35.7)	1.000	**0.553**
Male	31(67.4)	9(64.3)	0.900	
**Tribe**				
Ganda	28(60.9)	9(64.3)	1.119	**0.151**
Others	18(39.1)	5(35.7)	1.000	
**Marital status**				
Married	34(73.9)	9(64.3)	1.000	**0.0001**
Single	12(26.1)	5(35.7)	1.405	
**Occupation**				
Unemployed	14(30.4)	7(50.0)	1.000	**0.066**
Employed	32(69.6)	7(50.0)	0.538	
**Level of education**				
Below primary level	19(41.3)	8(57.1)	1.000	**0.306**
above primary level	27(58.7))	6(42.9)	0.614	
**Smoking status**				
Yes	4(8.7)	2(14.3)	0.150	**0.015**
No	42(91.3)	12(85.7)	1.000	
**Alcohol consumption**				
Yes	22(47.8)	3(21.4)	1.000	0.001
No	24(52.8)	11(78.6)	2.619	
**Presenting Concerns (days)**				
<3	26 (56.5)	9 (64.3)	1.000	0.591
>3	20 (43.5)	5 (35.7)	0.778	
**Temperature**				
Afebrile	37 (80.2)	10 (71.4)	1.000	0.474
Febrile	9 (19.6)	4 (28.6)	1.446	
**GCS**				
15	14 (30.4)	2 (14.3)	1.000	0.124
<15	32 (69.4)	12 (85.7)	2.182	
**Blood pressure**				
SBP>140	21 (45.7)	4 (28.4)	1.000	0.222
SBP 90-<140	25 (54.3)	10 (71.4)	1,786	
**HIV**				
Reactive	4 (8.7)	2 (14.3)	1.444	0.690
Non-reactive	40 (86.9)	12 (85.7)	1.000	
**Disability**				
0–3	13 (28.3)	3 (21.4)	1.000	0.136
4–6	33 (71.7)	11 (78.6)	1.333	
**Stroke severity**				
0–15	25 (54.3)	3 (21.4)	1.000	0.095
16–42	21 (45.7)	11 (78.6)	3.208	
**Type of stroke**				
Haemorrhagic	20 (43.5)	6 (35.7)	1.000	0.0001
Ischaemic	26 (56.5)	7 (64.3)	1.286	

**Table 4:** Multivariate analysis of factors associated mortality among young adult stroke patients at MNRH and KNRH.

Variable	adjusted PR	P-value	95% CI
**Smoking status**			
Yes	5.094		
No	1.000	<0.0001	3.712, 6.990
**Alcohol consumption**			
Yes	1.000		
No	7.247	<0.0001	4.491, 11.696
**Stroke severity**			
Minor& moderate	1.000		
moderate-severe &severe	3.301	0.007	1.395, 7.808
**Marital status**			
Married	1.000		
Single	0.785	0.110	0.583, 1.056
**Type of stroke**			
Haemorrhagic	1.000		
Ischemic	1.473	0.210	0.804, 2.700

PR- prevalence ratio, CI-confidence interval, WBC-white blood cells

## Discussion

We set out to determine the proportion of young adults, their associated factors and the 30-day mortality outcome and associated factors among young adults with stroke in our setting. Our results indicated that 31.3% of the stroke patients were young and 23.3% of them died within 30 days of suffering a stroke. Those who had ischemic strokes had a better survival probability compared to those with haemorrhagic strokes.

Our study reported a higher proportion of young adults compared to Tanzania (25.4%) [21], Morocco (28.9%) [22], West Africa (25.4%) [23] and high income countries (18.6%) [24]. The variations among similar African settings could be attributed to the differences in stroke risk factors profiles, population structures and referral systems and health care provision. The proportions are different from the high-income countries due to the high prevalence of stroke risk factors like sickle cell disease, rheumatic heart disease and high rates of alcohol abuse among the young adults [8, 9]. This may be an actual reflection of the global increasing occurrence of stroke in the young adults [4]. The increase may also be due to increased stroke awareness and increased health care services, increased access to health care, and improved diagnostics in the region. The high proportion rates of stroke in the young in the SSA may be due to the fact that there is a predominantly younger population in Uganda [25].

Targeted primary stroke preventive efforts are need to be further developed and utilized to stem this high burden.

About two-thirds of the young adults with stroke in this study had NIHSS severity scores of greater than 16 at admission. This is similar to an earlier study from Tanzania, where 43.9% of the young adult participants had severe stroke [21]. The severity of stroke may be associated with hospital delay where majority of patients came in with presenting complaints of about 3 days thus presenting late to hospital making time sensitive interventions like thrombolysis un-applicable. The delay in seeking timely medical care aligns with the fact that the majority of these patients were male, and it is known that males often exhibit poor health-seeking behaviours [26]. There is also lack of information regarding hospital seeking behaviours among young adults with stroke in Uganda to guide policy.

In this study we found a high 30-day mortality rate of 23.3%, however this is lower than Nigeria (23.9), Tanzania (49.1), Brazil (3.8), and Finland (15.7) [16, 21, 27, 28]. The mortality rate of 23.3% is higher than an earlier study done by eight years ago which reported a rate of 18.4% [14]. Lack of a comprehensive stroke centre and delays in seeking health care among this population might have played a role in these high mortality rates. Majority of the stroke patients had NIHSS >16 (moderate severe to severe stroke). After stroke onset, time is a crucial factor in acute ischemic stroke treatment with a higher probability of a successful outcome if the patient is treated within the first 90 minutes, however, majority of the patients sought care after three days. Health education and improving stroke knowledge is urgently needed especially early recognition of warning signs, such as FAST (facial drooping, arm weakness, speech difficulties and time to call) by the patient or immediate caregiver. Development of dedicated stroke centres to provide timely interventions to the patients in Uganda.

Whereas the results of this study provide useful insights and highlight the burden of stroke in the young adults in Uganda, they are based on small in-patient numbers should be interpreted with caution. As the more severely affected patients are more likely to die at home or might be referred to other nearby hospitals hence making it difficult to generalized the findings from these two specialized hospitals. The unfavourable patient factors such as lack of knowledge regarding hypertension, lack of specialized stroke centres and resources in settings where patients have no health insurance cover remains a major barrier in addressing stroke mortality and morbidity in the young adults.

### Limitations

This study had several limitations. First, due to being a hospital-based study, we might have missed a substantial number of patients with strokes as only those with severe symptoms might have been referred due to survivorship bias. Also, these findings may not be generalizable to the general population. Consecutive sampling methods has selection bias in which a variable that is associated with the outcome under investigation may occur more frequently or less in those sampled in this period as compared to the general population. Thirdly, we were unable to provide more details related to the duration of hypertension, treatment regimen followed among the study participants, as no corroborating history for hypertension duration either through the immediate next of kin or medical documents were availed. We also note that the history was mostly collateral and details of the history of hypertension were not fully obtained. The sample size was small to detect all but the strongest associations with common exposures. There were challenges of assessing physical activity coupled with inconsistencies from the responses hence the results were excluded from the data analysis. This therefore limited the statistical analysis especially when examining associations and other important prognostic factors. A well-designed study with a large sample size might be needed to explore this.

### Recommendations

Further study of the relationship between alcohol and stroke mortality outcome. Increase screening and management for stroke risk factors, especially hypertension, smoking and alcohol consumption among young adults. Rehabilitation services should be availed to alcohol consumers. Further studies to determine the drivers of high prevalence of haemorrhagic stroke and stroke severity.

## Conclusion

A third of all stroke patients were young adults with almost half with haemorrhagic stroke type, and a third with severe strokes. The 30-day mortality outcome was high and was associated with smoking, not drinking alcohol and a high NIH stroke score greater than 16. Primary and secondary stroke preventive interventions need to be enhanced to reduce stroke burden among young adults in Uganda.
